# Epitope Mapping of HIV-Specific CD8+ T cells in a Cohort Dominated by Clade A1 Infection

**DOI:** 10.1371/journal.pone.0006965

**Published:** 2009-09-11

**Authors:** Lyle R. McKinnon, Xiaojuan Mao, Joshua Kimani, Charles Wachihi, Christina Semeniuk, Mark Mendoza, Binhua Liang, Ma Luo, Keith R. Fowke, Francis A. Plummer, T. Blake Ball

**Affiliations:** 1 Department of Medical Microbiology, University of Manitoba, Winnipeg, Canada; 2 Department of Medical Microbiology, University of Nairobi, Nairobi, Kenya; 3 National Microbiology Lab, Public Health Agency of Canada, Winnipeg, Canada; 4 National HIV and Retrovirology Laboratory, Public Health Agency of Canada, Ottawa, Canada; 5 Department of Immunology, University of Manitoba, Winnipeg, Canada; Karolinska Institutet, Sweden

## Abstract

**Background:**

CD8+ T cell responses are often detected at large magnitudes in HIV-infected subjects, and eliciting these responses is the central aim of many HIV-1 vaccine strategies. Population differences in CD8+ T cell epitope specificity will need to be understood if vaccines are to be effective in multiple geographic regions.

**Methodology/Principal Findings:**

In a large Kenyan cohort, we compared responsive CD8+ T cell HIV-1 Env overlapping peptides (OLPs) to Best Defined Epitopes (BDEs), many of which have been defined in clade B infection. While the majority of BDEs (69%) were recognized in this population, nearly half of responsive OLPs (47%) did not contain described epitopes. Recognition frequencies of BDEs were inversely correlated to epitopic sequence differences between clade A1 and BDE (*P = *0.019), and positively selected residues were more frequent in “new” OLPs (without BDEs). We assessed the impact of HLA and TAP binding on epitope recognition frequencies, focusing on predicted and actual epitopes in the HLA B7 supertype.

**Conclusions/Significance:**

Although many previously described CD8 epitopes were recognized, several novel CD8 epitopes were defined in this population, implying that epitope mapping efforts have not been completely exhausted. Expansion of these studies will be critical to understand population differences in CD8 epitope recognition.

## Introduction

The immunodominance of T cell responses, or the relative proportion of the overall response attributable to any one given epitope, is governed by many factors[1]. While most intracellular pathogens are processed in the cytosol into thousands of peptides, only a few of these will generate epitope-specific CD8+ T cell responses. Successful epitopes must “pass” several steps before generating a response, including proteosomal cleavage, stability in the cytosol, ability to bind Transporter associated protein (TAP) and Human leukocyte antigen (HLA) alleles, and finally recognition by an appropriate T cell receptor (TCR). For pathogens such as human immunodeficiency virus (HIV), in addition to the above factors, the repertoire of available epitopes may critically differ between different viral strains.

In HIV infection, CD8+ T cell responses can be very broad and large in magnitude; in clade B-infected cohorts, >60 distinct epitope-specific responses have been measured in a single chronically infected subject[2], and the total HIV-specific CD8+ T cell response can be >15% of circulating CD8+ T cells in blood[3]. It has further become evident that not all of these responses are important for control of viral replication and prevention of disease[3], [4]. A better understanding of factors that influence CD8+ T cell recognition in HIV infection, particularly in the setting of different HIV clades, is of critical importance for HIV-1 vaccine efforts.

The cross-reactivity of HIV-specific CD8+ T cell responses has been addressed in several populations, which have found modest degrees of cross-clade reactivity, with some clade-specific responses [5], [6], [7], [8], [9]. Incomplete cross-reactivity poses a problem for the accurate assessment of HIV-specific CD8+ T cell responses, as a portion of responses will be missed due to differences between the sequence used for screening and the one that primed an individual’s responses *in vivo*. Consensus sequences are biased, as by definition these are mismatched to an individual’s autologous quasispecies. OLP libraries based on autologous sequences demonstrated that >30% more responses can be detected when compared to a consensus OLP library[10]. However, this approach is costly and impractical for screening large populations.

The optimal methods for defining HIV-specific CD8+ T cell responses continue to be debated. In addition to assessing multiple functional parameters[11], an important consideration is selection of the overlapping peptide (OLP) library sequence that best accounts for the genetic diversity of HIV-1, and matches the HIV-1 strains that are circulating in the study population. Because HIV-1 often varies or “toggles” between two amino acids at a given position, inclusion of these toggle peptides in combination was demonstrated to increase detection frequency[12]. Another strategy to increase detection sensitivity and account for HIV-1 diversity has been the use various clade or group M ancestral sequences, which phylogenetically share similarity with a greater number of circulating strains than any given circulating sequence itself, and this has been met with mixed success for increasing sensitivity[13], [14]. One report suggested that clade C peptides were better than clade B peptides at detecting reponses in a clade B-infected population[15].

Most of these strategies detect overlapping sets of HIV-specific T cell responses, yet the extent to which epitope recognition between populations differs, particularly where circulating strains and HLA allele frequencies differ, remains in question[16]. Other approaches to characterize the breadth and specificity of CD8+ T cell responses include the use of epitope prediction algorithms and previously defined epitopes, of which many have been defined in HIV-infected subjects. However, the majority of HIV research until recently has focused on clade B, which accounts for only a small percentage of HIV-1 infections globally[17]. Therefore, previously described HIV epitopes are biased towards well-studied populations that do not represent the majority of HIV-infected subjects, and studies that compare epitope recognition between populations are important to guide the design of vaccines that aim to be efficacious in multiple populations.

We assessed factors influencing HIV-1 Env CD8+ T cell epitope recognition in a large Kenyan population, where clade A1 comprises the majority of circulating sequences. A variety of factors known to influence epitope recognition, including HLA restriction, frequency, and peptide binding capabilities, HIV genetic diversity, and processing-related factors (TAP binding, proteosomal cleavage), were investigated. We also determined whether recognition of particular Env epitopes was associated with altered disease progression. These data demonstrate that while substantial overlap existed between the epitopes recognized in our study and those previously described, there were numerous instances of clade-specific epitopes. A better understanding of how CD8+ T cell responses differ in populations infected by different HIV clades has important implications for the design of cross-clade HIV vaccinogens.

## Materials and Methods

### Study subjects

Study subjects were enrolled in this study from a well-characterized female sex worker cohort based in Nairobi, Kenya. All gave informed consent to participate in this study, which was approved by the Institutional Review Boards at Universities of Manitoba and Nairobi. Subjects in this longitudinal cohort are sampled every 6 months, and CD4 counts measured for calculating disease progression. To assess various stages of disease progression, we calculated the number of years from enrollment until consecutive CD4 levels dropped below 200, 350, and 500 cells/ul of blood, and below 15% of T cells. At least two CD4 counts below each cut-off were used to decrease the influence of transient decreases. Subjects who did not progress during ≤5 years of follow-up, and those who progressed after a gap in follow-up of ≥3 years were excluded from this analysis. **All subjects were HLA typed using a taxonomy-based sequencing method, as described elsewhere [18].**

### Peptides and IFN-γ Elispot assays

The overlapping peptide (OLP) library used in this study was comprised of 158 15mers overlapping by 10 amino acids, assembled into 26 pools, was used to screen peripheral blood mononuclear cells (PBMC) of HIV-infected subjects for Elispot responses (*n = *61). **The sequence used for the OLP library was a clade A1 isolate from Uganda (Accession no. U15119).** Elispot assays were conducted as previously described[19]. 96-well nitrocellulose plates were coated with primary IFN-γ monoclonal antibody (Mabtech), and blocked with RPMI 10% fetal bovine serum (R-10). Freshly isolated PBMCs were incubated overnight at 2×10^5^ per well in duplicate with peptide pools, with each peptide at 3 ug/ml, or with individual peptides at 10 ug/ml. Plates were developed the following day using Mabtech and Biorad reagents, and dried plates were counted on an automated Elispot reader. Positive responses were defined as those more than double negative control and ≥50 spot-forming units (SFU) per million PBMCs. To be conservative in estimating response breadth, responses to 2 overlapping peptides were called one response; to 3 overlapping peptides were called 2 responses.

### Analysis of positive selection

Positive selection in HIV-1 Env was determined using sequences in the Los Alamos HIV Sequence database and the QUASI program[20]. QUASI is a codon-based method that measures empirical dN/dS ratios at each codon and compares these statistically to neutral dN/dS ratio using two-binomial distribution. The null hypothesis is that all mutations on a given codon should be equal (dN/dS = 1). If non-synonymous substitutions are more abundant than expected, the null hypothesis is rejected, and positive selection is determined to be occurring at that site. QUASI is independent of phylogeny and appropriate for large-scale (>100) sequence analysis.

### ITOPIA™ HLA binding assays

These assays were carried out using the iTopia Epitope Discovery System Kit (Beckman Coulter). These assays utilize microtitre plates pre-coated with the B*0702 MHC monomer folded correctly via β_2_ microglobulin (β_2_M) and a placeholder peptide. Peptides of interest (9mers), including positive controls, were resuspended in DMSO at [10 mM], and diluted 1∶90 in renaturation buffer prior to use. Stripping buffer was used to remove placeholder peptide and β_2_M, followed by washing three times with wash solution and immediate addition of renaturation mixture (renaturation buffer, anti-HLA-ABC-FITC conjugated monoclonal antibody, β_2_M and ddH_2_O). Diluted peptide was added and incubated for 18 hrs at 20°C with continuous, gentle shaking. Plates were then washed 3 times with wash solution, wells resuspended in dilution buffer, and read in a fluorimeter. Data was analyzed using iTopia software, and presented as percentage of positive control peptide binding.

### Statistical analyses

We used Fisher’s Exact Test to determine *HLA*-OLP associations (and Boneferoni test for multiple comparisons), Spearman’s Rank correlation to determine associations between response frequency and HIV diversity, and Mann Whitney tests to determine differences between groups of continuous variables.

## Results

**Figure 1:**
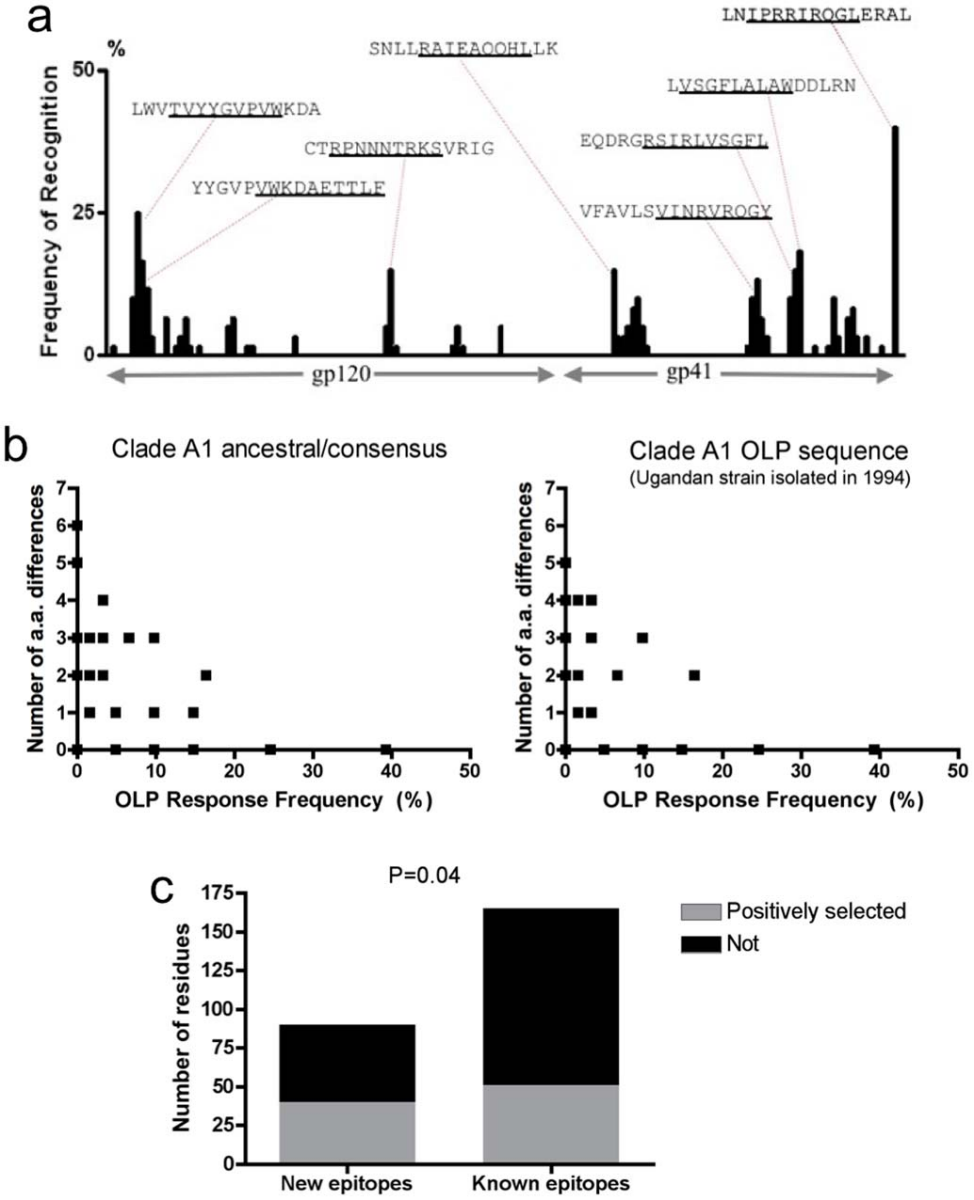
Influence of HIV diversity on CD8+ T cell epitope recognition frequencies. Epitopes recognized by a Kenyan cohort (n = 61) are shown, with the sequence of the most frequent optimal OLP sequences indicated and the optimal length epitope underlined a). Inverse relationship between OLP Elispot response frequency (x-axes) and the number of amino acid differences between BDE and ancestral/consensus clade A1 sequence (left panel) and clade A1 OLP library sequence (right panel) b). Positive selection was more commonly observed in new epitopes compared to BDEs c).

We measured IFN-γ Elispot responses to Env OLPs in a large HIV-infected cohort (*n = *82). For the majority of subjects, Env pool-specific responses were confirmed at the individual peptide level (*n = *61). A total of 215 peptide-specific responses were detected, and 34% of Env OLPs (53/158) were recognized at least once. After correcting for overlapping responses (see Methods), 165 epitope-specific responses were measured (mean 2.7 **Env** peptides per subject, *n = *61). Clustering of epitopes was evident, with the majority of epitopes found in the more conserved regions of HIV-1 Env (Fig. 1a), as has been described previously[21]. Despite being a smaller protein, gp41-specific responses were greater in frequency as compared to gp120-specific responses (133 responses to 58 peptides vs. 82 responses to 100 peptides, 2.29 vs. 0.82 responses/OLP, p<0.0001, Chi-square test). **One possible explanation for these data is that gp41 is more conserved than gp120, and therefore is a closer match to the OLP sequence used for screening.** The mean and median magnitudes of responses were 583 and 363 SFU/million PBMCs, respectively (range 53–3578 SFU/million).

Given that there are several well-defined epitopes for HIV-1 Env, we compared the OLPs that elicited responses in our study with the list of *Best Defined Epitopes* (BDEs) for Env published in the Los Alamos HIV Immunology database[22]. Many BDEs were frequently targeted in our study, often at high magnitudes, confirming that the *Best Defined Epitope* list is relevant in this divergent population. The majority of BDEs (69%, 18/26) were targeted by ≥1 subject in our study, 54% (14/26) by ≥2 subjects, and 32% (9/26) by ≥3 subjects. However, there were several instances of BDEs that were not recognized by the subjects in our OLP screen (Table S1), and several responses to OLPs that have not previously been shown to contain BDEs (Table 1).

**Table 1:** OLPs targeted in the current study (by ≥2 subjects) that are not found on the Best Defined Epitope (BDE) list.

OLP Sequence aligned to clade B consensus (below)	Overlapping Peptide (OLP)	Env position (HXB2)	No. of Responders (n = 61)	Previous descriptions of epitopes in the region
**LVSGFLALAWDDLRN** **----D---------I-------------**	OLP-149	749–63	11 (18.0%)	20mer (742–61) includes this epitope, no HLA
**GRSIRLVSGFLALAW** **D---G-----D---------I----**	OLP-148	744–58	9 (14.8%)	
**EQDRGRSIRLVSGFL** **--R----D---G-----D-----**	OLP-147	739–53	6 (9.8%)	
**ELLGHSSLKGLRLGW** **-------deleted.............**	OLP-156	783–97	6 (9.8%)	786–794 (different sequence)
**LGNLLLYWGRELKTS** **WW---Q-----SQ-----N-**	OLP-160	796–810	5 (8.2%)	799–807, different epitope
**IKQLQARVLAVERYL** **----------------------------**	OLP-114	573–87	5 (8.2%)	19mers (570–589, 572–590)
**EVHNVWATHACVPTD** **------------------------------**	OLP-13	64–78	4 (6.5%)	described as 20mer (62–80), no HLA
**VRQGYSPLSFQTLTP** **-----------------------RL--**	OLP-141	708–22	4 (6.5%)	nothing
**IYMENVTEEFNMWKN** **VVL---------N-------------**	OLP-17	84–98	4 (6.5%)	A*11, A*68, clade B version
**NITNNITNSITNSSV** **-----TS--RDKVQKEYA**	OLP-27	160–74	4 (6.5%)	nothing
**LTVWGIKQLQARVLA** **-----------------------------**	OLP-113	568–82	3 (4.9%)	see OLP-114
**LDCSYNITNNITNSI** **KN----F------TS--RDKV**	OLP-26	155–69	3 (4.9%)	156–65 Cw8
**QHLLKLTVWGIKQLQ** **--------Q-------------------**	OLP-112	563–77	2 (3.3%)	565–573 (A2)
**AIEAQQHLLKLTVWG** **-----------------Q----------**	OLP-111	558–72	2 (3.3%)	closest is 557–65 w R in front
**PNPQEIYMENVTEEF** **---------VVL----------N-**	OLP-16	79–93	2 (3.3%)	78–86 B*3501, w D in front
**SPLSFQTLTPNPRDP** **-------------RL-A----G--**	OLP-142	713–27	2 (3.3%)	nothing

Optimal epitopes = underlined.

### Influence of HIV genetic diversity on epitope recognition frequencies

Many factors could explain discordances between OLPs recognized in our study and the BDE list. For example, sequence differences between the clade A1 (used in our OLP library) and the BDE (in most cases clade B) could result in clade-specific epitope targeting. We calculated the number of differences between BDEs and the corresponding epitope sequence of the OLP library, and found an inverse correlation between the number of amino acid differences and the frequency at which that OLP was recognized (Fig. 1b, r ^2^ = −0.457, *P = *0.019). A similar inverse correlation was observed between number of amino acid differences (consensus or ancestral A1 vs. BDE) and OLP recognition frequency (r^2^ = −0.399, *P = *0.043), demonstrating that sequence differences between clades A1 and B correlate with less frequent recognition of BDEs in a clade A1-infected population. For example, only one of the commonly recognized epitopes (OLP-8, recognized by >10% of subjects) had substantial amino acid differences (≥2) between clade A1 and B (Table S1). Conversely, for peptides such as OLP-62, where six amino acid differences exist between the OLP sequence and BDE, it is not surprising that this BDE was not recognized in clade A1-infected subjects (Table 1). These data suggest that conserved BDEs are recognized at a higher frequency than BDEs that vary in sequence from clade A1.

Since the majority of epitopes cluster in conserved regions of HIV-1 Env (Fig. 1a), we next compared the frequencies of positively selected residues within epitopic and non-epitopic regions. Positive selection, as determined by comparing the rate of non-synononous over synonomous mutations, is indicative of population level selection pressure at any particular residue, and also regions of the HIV genome that more readily accommodate sequence variation. Prior analyses of HIV sequences from the Los Alamos HIV Seqeunce database demonstrated evidence of positive selection at 288 of 856 Env amino acid residues[20]. Including only OLPs recognized by >5% of our study population (≥4 subjects, *n = *17), no differences in positive selection were apparent between residues located inside versus outside of responsive OLPs (34 vs. 33%). We next compared selection within OLPs that contained or lacked BDEs, and found that responsive OLPs without BDEs had a higher rate of positively selected residues compared to those containing BDEs (44% vs. 29%, *P = *0.04, Fig. 1c). Given our data that sequence differences can have a negative impact on recognition frequency, these analyses suggest that new epitopes might not have been recognized previously because they lie in regions of HIV that are less evolutionarily constrained and therefore easily escapable, removing potential epitopes from circulation in a given population.

Although sequence conservation within an OLP increased its recognition frequency, there were examples where sequence difference alone appeared to have less impact on CD8 recognition. The most obvious examples were OLPs that were identical between clades A1 and B, yet have no epitopes described to date (i.e. OLP-13, 113, 114; Table 1). There were also examples of OLPs containing a BDE that were targeted in spite of sequence differences (i.e. OLP-139). This could be because sequence differences occurred in the TCR recognition portion of the epitope, leaving epitope-HLA binding intact (Table S1). Retention of HLA binding has been shown previously to be an important predictor of cross-clade reactivity, presumably because an appropriate TCR can be induced as long as the epitope is still presented[7]. The reasons why intact epitopes were recognized by this population and not those studied previously, despite HLA similarities, were less clear. It is important to note that many OLPs containing intact BDEs (ie, OLP-7, 110, and 169, Table S1) were frequently recognized, cross-clade epitopes in this cohort, which could be useful in HIV vaccines.

### Clade A1-specific epitopes recognized in our OLP screen

**Figure 2:**
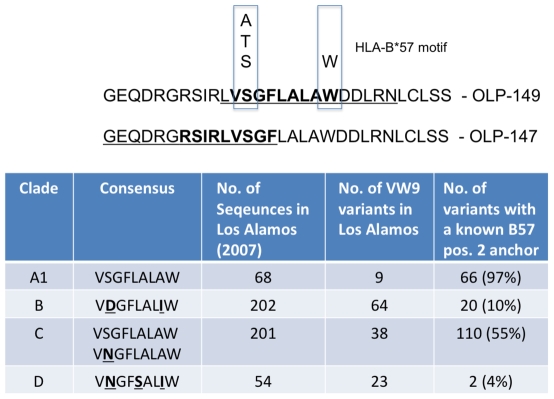
OLP-147 and 149 represent clade A1-specific epitopes. These OLPs associate with HLA-B*57 expression (Table 2) and with the anchor residues that have been defined for the HLA-B*57 binding motif a). Alignments of the optimal epitope within OLP-149 demonstrate that in other clades, this epitope would not likely be presented by HLA-B*57 b).

We observed some examples of frequently recognized epitopes that appear to be clade A1-specific. OLPs-147, 148, and 149 were frequently recognized and contain no described epitopes to date. These OLPs were weakly associated with HLA-B*57 expression, but these associations did not survive multiple test comparisons (described below, Table S2). Two potential epitopes within these OLPs, VSGFLALAW and RSIRLVSGF, fit the HLA-B*57 binding motif (Fig. 2a), and we confirmed these as recognizable epitopes in Elispot assays using PBMCs from HLA-B*57+ subjects (not shown). These responses were often immunodominant; OLP-149, where targeted, was typically the strongest Env-specific response, and pool 23 (containing OLP147, 148, and 149) was the dominant Env pool in >75% of B*57+ subjects tested (not shown). The clade B version of this epitope contains aspartic acid at position 2 in the epitope (Fig. 2b). Because the HLA-B*57 binding motif prefers alanine, threonine, or serine as position 2 anchors, this epitope does not appear to be available in clade B, providing a possible explanation as to why it has yet to be described. Given the additional differences in clades C and D in this region, it is likely that these immunodominant epitopes are clade A1-specific.

OLP-156 is another example of a commonly recognized pepitde in our population (9.8% of subjects) with no decribed epitopes to date (Table 1). Sequence alignments of this OLP with other clades demonstrated that while relatively conserved in clade A1, a seven amino acid stretch is absent in most sequences from clades B and D, and is substantially different in clade C (not shown). Therefore, OLP-156 is another example of a clade A1-specific epitope.

### Influence of HLA on epitope recognition frequencies

Differences in HLA frequencies between populations may also contribute to differences in CD8+ T cell epitope recognition frequencies. Only 15% of responses to OLPs containing BDEs (16/104) were made by subjects with a previously described HLA restriction for that BDE. This phenomenon of multiple alleles presenting the same epitope has been referred to as cross-restriction. Therefore, 85% of responses detected in this cohort represent new epitopes and/or new HLA restrictions of described epitopes. Although the inclusion of HLA supertypes would increase the number of responses with putative restrictions, this analysis must be approached with caution given that subtle differences exist between the epitope restrictions of closely related alleles [23]. Overall, there was no correlation between the frequency of HLA alleles known to present BDEs within a 15mer OLP and the response frequency to that OLP (p = 0.6, not shown). These data suggest that subjects are either recognizing different epitopes within an OLP, or more likely that epitope recognition commonly occurs in the context of multiple HLA alleles.

There were several examples that illustrated how HLA frequency and/or HIV-1 diversity can influence epitope recognition frequency. OLP-82, which was not recognized despite being conserved between clades A1 and B, contains two epitopes restricted by HLA alleles (B*5101 and A*3201) that are present at low frequencies in our cohort (3 and 3.7%). Therefore, even though the epitope sequence was available, the HLA alleles needed to present the epitope were infrequent. Conversely, some BDEs restricted by common alleles (>10% in frequency; i.e. HLA-A*3002-HIGPGRAFY and A*0201-RGPGRAFYVTI) had several sequence differences between clades A1 and B which could explain their lack of recognition in our study (Table S1). Finally, for the HLA-A*3101-restricted epitope RLRDLLLIVTR, a low cohort HLA frequency (<2%) combined with several sequence differences correspond to lack of recognition of this OLP in our study. These data suggest that HLA allele frequency or HIV-1 diversity, and in some cases both, can limit the availability of BDEs in another population.

We next measured associations between responses to an OLP and expression of specific HLA alleles by those subjects. We restricted these analyses to HLA alleles expressed by ≥5 subjects (*n = *36), and OLPs recognized by ≥4 subjects (*n = *19), for a total of 684 comparisons. These analyses also included data from a recently published report[24] (n≥90 tested for most OLPs). We observed 44 significant associations at the P<0.05 level, and 20 at P<0.01 (Table S2). Only five associations between OLP recognition and HLA allele were significant after taking multiple comparisons into account (*P<*0.0001, Boneferoni test). The most frequently recognized peptide in this population was OLP-169, recognized by 32% of subjects tested (n = 99, not shown). OLP-169, which contains the B*0702-restricted BDE IPRRIRQGL, was recognized by 83% (20/24) of HLA-B*4201+ subjects, compared to 16% (12/75) of B*4201-negative subjects (P = 2.96×10^−9^). Similarly, 88% (7/8) of B*0702+ subjects recognized OLP-169, while only 27% (25/91) HLA-B*0702-negative subjects responded to this peptide (P = 0.001). Together these B7 supertype alleles appear to restrict the majority of responses to OLP-169, which was the immunodominant CD8 epitope in this population.

### Predicted versus recognized epitopes for the B7 supertype

Among the methods proposed for the definition of CD8 epitopes, a number of epitope prediction algorithms have been employed to decrease the number of potential epitopes needing confirmation in immunological assays. We therefore evaluated the ability of these algorithms to predict which epitopes would be targeted in our epitope mapping study. Using motifs that have been defined for seven HLA supertypes[25] and the clade A1 OLP library used in our study, a wide range of epitopes were predicted, depending largely on how narrowly each HLA motif had been defined (this included 10 epitopes for HLA-B7, 28 for B44, 26 for A1, 149 for A2, 28 for B27, 115 for A24, and 48 for B58).

We focused our analysis on the HLA-B7 supertype given its immunodominance in our epitope mapping and association data. Of the 10 predicted epitopes, only one was recognized in this study (IPRRIRQGL, contained in OLP-169). Another B7 epitope that was frequently recognized (OLP-60), RPNNNTRKSI, was predicted when the analysis was extended to 10mers. Very similar results were observed when the predictions were repeated with clade B Env; all 10 epitopes were predicted, plus an additional 3 not predicted for clade A1, suggesting a similar set of epitopes predicted between clades.

We next examined possible reasons why most predicted epitopes were not targeted by CD8+ T cells in our population. We hypothesized that predicted epitopes could go unrecognized where the autologous HIV sequences in subjects tested differed from the sequence of the OLP library. We examined the autologous quasispecies of subjects who possessed a B7 supertype allele but tested negative for 9/10 of the predicted epitopes (*n = *4). Sequence differences between autologous and OLP library were common in these subjects (Table 2). All of these differences were observed in non-HLA anchor residues, suggesting that the residues required for HLA binding remained intact in the autologous sequences of these subjects. Although we did not test these variants for T cell responses, it appears that the predicted epitopes were at least available for HLA presentation in HLA-B7+ subjects who nevertheless did not recognize them.

**Table 2:** Predicted epitopes based on the *HLA-B7* supertype motif and the clade A1 OLP library sequence used for epitope mapping.

U15119 position	Seqence	TAP binding scores*	ITOPIA HLA-B*0702 binding (%)**	Autologous Seq.***
**8–16**	YPCWWTWGI	2.893	none	no data
**75–83**	DPNPQEIYM	−0.901	none	DPNPQEIELDPNPREISLDPNPQEIPLDPNPQEVVL
**114–22**	KPCVQLTPL	2.175	64.2(----K----)	KPCVKLTPL KPCVQLTPLKPCVKLTPLKPCVKLTPL
**205–13**	CPKVTFEPI	−1.025	30.1	CPKVNFEPICPKVTFEPICPKVSFEPICPKVTFEPI
**211–9**	EPIPIRYCA	2.869	none	EPIPIHYCAEPIPIHYCAEPIPIHYCAEPIPIHYCA
**213–21**	IPIRYCAPA	0.198	58.5(---H-----)	IPIHYCAPAIPIHYCAPAIPIHYCAPAIPIHYCAPA
**252–60**	RPVVSTQLL	1.393	78.1	KPVVSTQLLKPVVSTQLLKPVVSTQLLKPVVSTQLL
**408–16**	LPCRIKQII	2.046	75.9	no data
**489–497**	APTKAKRRV	2.482	95.2	no data
**843–51**	IPRRIRQGL	3.59 (intermed)	180.2	no data

Only the last epitope (in bold) was recognized.

* TAP binding scores were calculated using TAPpred, as per Ref. 20.

** Percentage of Positive control binding. Where ITOPIA-tested sequences differed from OLP, these are noted.

*** Autologous sequences are from HLA B7 supertype+ subjects who did not recognize the epitopes listed (except IPRRIRQGL). Each sequence = 1 subject.

To determine experimentally whether predicted epitopes were capable of binding HLA-B*0702, we tested these peptides in the ITOPIA™ *in vitro* HLA-peptide binding system. These experiments showed that 7/10 of the predicted epitopes bound HLA-B*0702, as demonstrated by ≥30% binding as compared to proprietry control peptide (Table 2). Of the predicted B7 epitopes, it was the frequently recognized IPRRIRQGL epitope (OLP-169) which bound to HLA-B*0702 with the strongest affinity (180% of positive control peptide). Therefore, because several predicted epitopes were positive for HLA binding *in vitro*, the lack of recognition of these peptides in our cohort (and others) could be because a) this binding is suboptimal for generating responses when competing with other antigen-specific responses, b) these responses have been lost through the course of infection, or c) these epitopes may not be readily processed or recognized by an appropriate TCR.

We addressed the latter hypothesis using bioinformatics tools that determined the TAP binding affinity[26] and proteasomal cleavage scores[27] of predicted B7 epitopes in the context of the clade A1 OLP library sequence. While all of the predicted but unrecognized epitopes had low/undetectable TAP scores (mean 1.35, Table 2), IPRRIRQGL had an intermediate TAP score of 3.59, comparable to the mean TAP score of Env BDEs (3.67). TAP scores did not correlate with the frequency that OLPs were recognized in our cohort (p = 0.3). There were no significant differences between TAP scores or proteosomal cleavage sites in clade B consensus as compared to the A1 OLP library in the overall data set. However, for certain epitopes TAP binding might influence clade-specific recognition frequencies. For example, the clade A1 version of EVAQRAYRA (EVGQRLGRA), recognized by only one subject, had a lower TAP binding score (2.78) as compared with the BDE (5.11).

### OLP responses are associated with altered disease progression

**Figure 3:**
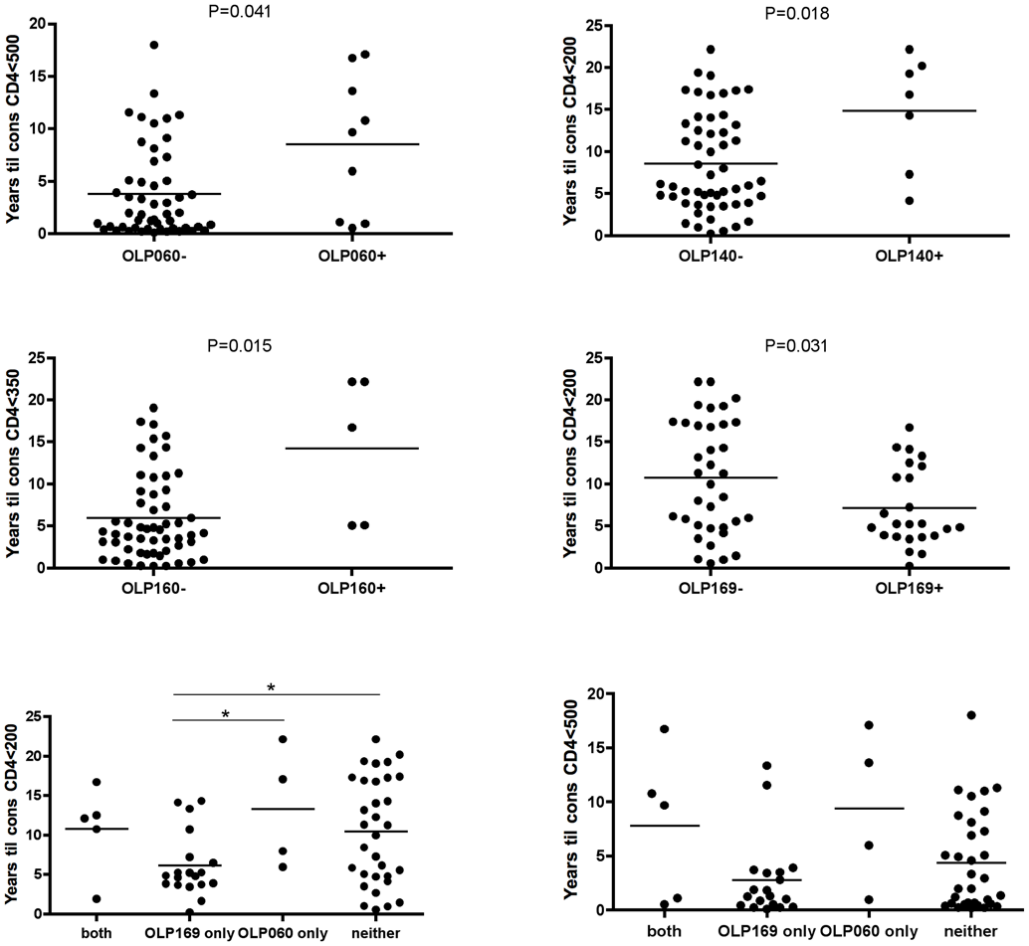
Recognition of certain OLPs was associated with clinical progression status. Three OLPs were associated with slower progression, and one with faster progression a). Recognition of OLP-060 and -169 in combination and implications for disease progression b).

To determine if recognition of any OLP was associated with altered progression to various stages of HIV disease progression, we compared the time to CD4 decline below 500, 350 and 250 in responders and non-responders for 17 OLPs recognized by ≥4 subjects (*n = *61, see methods). Responses to three OLPs were associated with slower progression to AIDS, and responses to one OLP with faster progression to AIDS (Fig. 3). OLP-60, -140, and -160 responders progressed approximately 2–3 times slower than non-responders to CD4 counts below 500, 200, and 350, respectively (p<0.05, Mann Whitney, Fig. 3a). In contrast, OLP-169 recognition was associated with an approximate two-fold faster time to consecutive CD4 counts below 200 (p = 0.031). Because the same subjects frequently recognized both OLP-60 and -169, we next compared the effect of recognizing these protective and detrimental OLPs in combination. Subjects recognizing both, neither, or OLP-60 only all progress more slowly to CD4<200 compared with those recognizing OLP-169 only (Fig. 3b). Similar data were observed for CD4<500. Interestingly, while both HLA-B*0702 and B*4201 associated with OLP-169 recognition, the related allele B*8101, which is the B7 allele most strongly associated with lower viral loads in other studies[28], was not associated with OLP-169 recognition (*P = *0.8). While causation cannot be inferred in a cross-sectional study, these data suggest recognition of certain OLPs may be beneficial or detrimental in the setting of chronic HIV infection.

## Discussion

The genetic diversity of HIV-1 is a major obstacle to both an accurate measurement of host immune responses and the design of successful HIV-1 vaccines. Historically, HIV-specific CD8+ T cell responses have been defined primarily in clade B-infected subjects, which account for a small minority of the global HIV-1 pandemic. We evaluated HIV-specific CD8+ T cell responses in a Kenyan cohort primarily infected with clade A1 (>75%)[29]. The majority of epitopes on the *Best Described Epitope* list were indeed recognized by these subjects (69%, 18/26). Exceptions were found in either direction; several BDEs were not recognized in our cohort (*n = *8, Table 1), and several responsive OLPs in our cohort did not contain BDEs (*n = *16, Table S1). The reasons for differences in epitope recognition between populations are likely numerous, given the number of host and viral factors that can influence recognition frequencies of CD8+ T cell epitopes. Our data suggest that sequence differences between BDEs and clade A1 may play a role in determining which BDEs were recognized. In addition, while many epitopes have now been defined, almost half of responsive epitopes (47%) recognized by ≥2 subjects in our study have yet to be described, suggesting that these efforts have not been entirely exhausted. This has been supported by a recent study which found that even for well studied HLA alleles, prediction software and *in vitro* immunological confirmations were able to define several new epitopes[30].

A wide array of host and viral determinants influence which epitopes result in immune responses. Host factors often depend on viral sequences, particularly where differences in viral sequence affect how an epitope is processed, presented, and recognized by T cells. Amino acid substitutions can be extra-epitopic, including those that affect epitope processing, or intra-epitopic, such as those that affect TAP binding, HLA binding, and TCR recognition. In the case of HLA-B7, we found that only one of the *in silico* predicted Env epitopes was frequently recognized in our Kenyan cohort. Many of the HLA-B7 subjects we tested possessed autologous sequences that differed from the clade A1 Env OLP library sequence, although the immunological consequences of these substitutions remain unknown. In addition, all of the predicted but unrecognized epitopes had low TAP binding scores, and others had low or undetectable binding to HLA-B*0702 *in vitro*. This suggests that while some of the predicted epitopes can bind HLA-B*0702, this binding may be sub-optimal *in vivo*, and moreover that other upstream factors may influence how well these predicted epitopes are loaded onto HLA and presented to T cells. This in turn would limit the number of epitopes for any given HLA allele at the population level. Because competition between T cell clones is believed to be an important determinant of immunodominance[31], better TAP and HLA binding could influence the amount of HLA-epitope that is available to stimulate a corresponding T cell response. The quality of TCR recognition is also likely to be critical, particularly the functional avidity of the HLA-epitope-TCR interactions. Previous work has demonstrated that HLA binding alone did not predict the recognition frequency of HIV and EBV epitopes[32].

These epitope mapping data corroborate our previous study in this population showing that while substantial cross-clade reactivity was present, there were also a subset of subjects who displayed a preference for clade A1[8]. This has since been supported by other studies of mixed clade infections[33]. The data presented here suggest that recognition of a combination of common and clade-specific epitopes provides a plausible explanation for our prior clade A1 preference data. We identified examples of novel, clade A1-specific epitopes (OLP-149, 156) that were recognized frequently in our cohort. Neither of these epitopes was available in a presentable form in most clade B (and other clade) sequences, and therefore subjects infected by clade B would not likely be exposed to these peptides, even though they could have HLA alleles capable of presenting, and TCRs capable of responding. These data suggest that each clade may have additional epitopes, not available in other clades, which could augment the breadth of HIV-1 vaccine responses in populations infected by these clades.

We found some associations between particular HLA class I alleles and responses to particular OLPs. Although our study was underpowered and therefore exploratory, other factors may impede the definition of HLA restriction by this method. For example, although OLP-7 was recognized by almost 25% of subjects, only one weak HLA association was observed (Table S2). A probable explanation for these data is that OLP-7 is promiscuous and can be presented by more than one HLA allele, as has been previously described[34], and therefore, a very large study would be required to identify its HLA restriction(s). Another instance where statistical power is lacking is the case where an epitope is not commonly recognized by subjects expressing the restricting allele. An example is the association between OLP-27 and B*1503; while all 4 subjects who recognized OLP-27 expressed HLA-B*1503, only 4/22 B*1503+ subjects responded to OLP-27 (*P = *0.005). Therefore, although HLA-B*1503 appears to be the presenting allele, it remains inconclusive without immunological confirmation.

Although Env-specific CD8+ T cell responses have previously been associated with higher viral loads, here we find that three Env epitopes associated with slower progression. While these are only associations and not proof of causation, it is possible that not all Env epitopes are detrimental in the setting of HIV infection. Conversely, the dominant Env epitope in this study (B7 supertype-restricted OLP-169) was associated with worse clinical outcome, raising the possibility that responses to certain epitopes can distract attention away from epitopes that are better capable of providing protection. One way to address this hypothesis would be to examine the relationship between protective and non-protective epitopes; if “bad” epitopes distract the immune system away from “good” responses, then one might predict there to be an inverse relationship, with a response to bad epitopes detracting from responses to good epitopes. Here we provide preliminary evidence that recognition of the protective epitope (OLP-060) may be advantageous even if the detrimental epitope (OLP-169) is recognized concurrently. These data will need to be confirmed in larger studies.

Previous studies have compared epitope recognition between HIV-infected populations. One report found that multiple ethnicities infected by clade B target similar immunodominant regions of HIV-1[2]. Clade C has also been the focus of some recent studies, including those in Southern Africa, India, and Ethiopia, all of which find similar epitope regions were targeted in respective clade C epidemics compared to clade B and other clade C-infected populations[35], [36], [37], [38]. Some instances of clade and region-specifc responses have also been observed. In China, where clade B/C recombinants dominate, clade B OLP responses tended to be higher magnitude when testing regions of HIV-1 that were predominantly clade B, and vice versa for clade C[39]. Our data also agree with another Kenyan study, which found that many CD8 responses in clade A were common to those found in clades B and C, and that additional OLP libraries do not appreciably increase the frequency of responses detected[40]. Therefore, although many responses are shared between clades, a consensus on the degree to which additional responses can be detected, and the clinical relevance of these responses, remains an important area of HIV vaccine research.

A more accurate definition of HIV-specific CD8+ T cell responses remains critical, both for assessment of HIV vaccine candidates and for a better definition of CD8+ T cell responses capable of delaying progression to AIDS. Here we have demonstrated that while the majority of BDEs were relevant in this Kenyan population, a number of additional OLPs without BDEs were frequently recognized. While sequence differences between clade A1 (dominant in Kenya) and B (focus of most studies) often appear to be capable of explaining these differences, a number of host factors also contribute to the recognition frequency of OLPs in our study. As a result, some important immunological targets may be clade-specific and therefore not detected in studies of other clades, even though they could remain useful for HIV-1 vaccine design. These data suggest that subtle differences in HIV-specific immunity exist between populations, with potential implications for approximating the breadth of vaccine coverage.

## Supporting Information

Table S1 Comparison of Best Defined Epitope (BDE) sequences with those in the overlapping peptide (OLP) library used in the current study.
(0.08 MB DOC)

Table S2 HLA class I allele-OLP associations. Statistical significance was determined using the Fisher's Exact test (P values shown are uncorrected).
(0.10 MB DOC)
