# Supplementary material for: Epitope Mapping of HIV-Specific CD8+ T cells in a Cohort Dominated by Clade A1 Infection
Source: PLoS One. 2009 Sep 11;4(9):e6965. doi: 10.1371/journal.pone.0006965 (PMC2735720; doi:10.1371/journal.pone.0006965)
Supplement: Table S1 — Comparison of Best Defined Epitope (BDE) sequences with those in the overlapping peptide (OLP) library used in the current study. (0.08 MB DOC) [file pone.0006965.s001.doc]

**Table S1.** Comparison of Best Defined Epitope (BDE) sequences with those in the overlapping peptide (OLP) library used in the current study.

| **BDE (top) versus OLP (bottom)** | **HXB2 Env** | **HLA*** | **Correpond. 15mer** | **BDE vs**  **OLP seq**** | **BDE vs**  **Cons A1 seq**** | **Responders N=61 (%)** |
| --- | --- | --- | --- | --- | --- | --- |
| ---------  LNIPRRIRQGLERAL | 843-851 | B*0702 | OLP-169 | 0 | 0 | 24 (39.3) |
| ----------  LWVTVYYGVPVWKDA | 037-46 | A*0301 | OLP-7 | 0 | 0 | 15 (24.6) |
| -----E-T--  YYGVPVWKDAETTLF | 042-51 | B*5501, B*3501 | OLP-8 | 2 | 2 | 10 (16.4) |
| ---------I  CTRPNNNTRKSVRIG | 298-307 | B*0702 | OLP-60 | 1 | 0 | 9 (14.8) |
| ----------  SNLLRAIEAQQHLLK | 557-565 | Cw*0304, Cw*15 | OLP-110 | 0 | 0 | 9 (14.8) |
| ----K----  ARVLAVERYLRDQQL | 584-592 | B*1402 | OLP-115 | 1 | 0 | 6 (9.8) |
| IV--N----  VFAVLSVINRVRQGY | 704-712 | A*3002 | OLP-139/140 | 3 | 3 | 6 (9.8) / 8 (13.1) |
| ----------  NTAENLWVTVYYGVP | 031-40 | B*4402 | OLP-6 | 0 | 0 | 6 (9.8) |
| --CW---Q-  EGLKYLGNLLLYWGR | 794-802 | A*3002 | OLP-159 | 3 | 2 | 4 (6.6) |
| ---K-----  VERYLRDQQLLGIWG | 585-593 | A*23, A*2402,  B*0801 | OLP-116 | 1 | 0 | 3 (4.9) |
| ---------  LEITTHSFNCGGEFF | 375-383 | B*1516, Cw*04 | OLP-74 | 0 | 0 | 3 (4.9) |
| --R.---A---  SSLKGLRLGWEGLKY | 786-795 | B*2705 | OLP-157 | 3 | 3 | 2 (3.3) |
| Q---N--VS-  LYWGRELKTSAINLF | 805-814 | B*4001 | OLP-161 | 4 | 4 | 2 (3.3) |
| -VI------  CNTSALTQACPKVTF | 199-207 | A*1101 | OLP-40 | 2 | 1 | 2 (3.3) |
| ------VVL  CVPTDPNPQEIYMEN | 078-86 | B*3501 | OLP-15 | 3 | 2 | 1 (1.6) |
| --E------  MHTDIISLWDQSLKP | 104-112 | B*3801 | OLP-21 | 1 | 1 | 1 (1.6) |
| --A--AY-  TDRVIEVGQRLGRAI | 831-838 | A*3303 | OLP-166 | 3 | 4 | 1 (1.6) |
| ------IV--  IGLRIVFAVLSVINR | 698-707 | A*3303 | OLP-138 | 2 | 2 | 1 (1.6) |
| S------H-  PKVTFEPIPIRYCAP | 209-217 | A*2902 | OLP-42 | 2 | 0 | 0 |
| H----RA--  SVRIGPGQTFYATGD | 310-318 | A*3002 | OLP-62 | 3 | 2 | 0 |
| R---RA-VTI  SVRIGPGQTFYATGD | 311-320 | A*0201 | OLP-62 | 6 | 5 | 0 |
| ---------  ITLPCRIKQIINMWQ | 416-424 | B*5101 | OLP-82 | 0 | 0 | 0 |
| ---------  ITLPCRIKQIINMWQ | 419-427 | A*3201 | OLP-82 | 0 | 0 | 0 |
| -A----A--  ICTTNVPWNSSWSNK | 606-614 | B*3501 | OLP-120 | 2 | 2 | 0 |
| R---LL--VT-  YHQLRDFILIVARTV | 770-780 | A*0301, A*3101 | OLP-153 | 5 | 4 | 0 |
| --T-I----  DFILIVARTVELLGH | 777-785 | A*6802 | OLP-154 | 2 | 3 | 0 |

* Previously described HLA restrictions for these epitopes

** Number of amino acid sequence differences between the sequences noted
