# Supplementary material for: Epitope Mapping of HIV-Specific CD8+ T cells in a Cohort Dominated by Clade A1 Infection
Source: PLoS One. 2009 Sep 11;4(9):e6965. doi: 10.1371/journal.pone.0006965 (PMC2735720; doi:10.1371/journal.pone.0006965)
Supplement: Table S2 — HLA class I allele-OLP associations. Statistical significance was determined using the Fisher's Exact test (P values shown are uncorrected). (0.10 MB DOC) [file pone.0006965.s002.doc]

**Table S2.** HLA class I allele-OLP associations. Statistical significance was determined using the Fisher’s Exact test (P values shown are uncorrected).

| OLP | Allele | Response+,  Allele+ | Response+,  Allele- | Response -,  Allele+ | Response -,  Allele - | P value |
| --- | --- | --- | --- | --- | --- | --- |
| 007 | A*3001 | 5 | 14 | 5 | 68 | 0.029 |
| 008 | A*2301 | 9 | 7 | 8 | 68 | 0.0002 |
| 008 | Cw*07 | 5 | 11 | 3 | 76 | 0.003 |
| 017 | A*3201 | 2 | 2 | 3 | 68 | 0.02 |
| 017 | B*5301 | 4 | 0 | 3 | 69 | <0.0001 |
| 027 | A*2301 | 3 | 1 | 10 | 63 | 0.014 |
| 027 | B*1503 | 4 | 0 | 18 | 56 | 0.005 |
| 060 | A*3402 | 2 | 10 | 1 | 85 | 0.05 |
| 074 | Cw*04 | 4 | 2 | 11 | 57 | 0.014 |
| 110 | A*66 | 5 | 9 | 5 | 76 | 0.006 |
| 110 | B*47 | 2 | 12 | 0 | 80 | 0.021 |
| 110 | B*4805 | 2 | 12 | 0 | 80 | 0.021 |
| 110 | B*8101 | 5 | 9 | 7 | 73 | 0.015 |
| 110 | Cw*0804 | 3 | 11 | 1 | 82 | 0.009 |
| 114 | A*6601 | 3 | 3 | 5 | 58 | 0.018 |
| 114 | B*5802 | 6 | 0 | 6 | 57 | <0.0001 |
| 114 | Cw*0602 | 5 | 1 | 16 | 48 | 0.008 |
| 115 | A*74 | 5 | 5 | 10 | 75 | 0.008 |
| 115 | B*14 | 4 | 6 | 3 | 81 | 0.002 |
| 115 | Cw*0802 | 5 | 5 | 2 | 85 | <0.0001 |
| 139 | A*3004 | 2 | 8 | 0 | 89 | 0.009 |
| 140 | A*3004 | 2 | 5 | 0 | 66 | 0.008 |
| 140 | A*7401/2g | 3 | 4 | 6 | 60 | 0.036 |
| 140 | B*3501 | 2 | 5 | 1 | 65 | 0.023 |
| 147 | A*74 | 4 | 5 | 9 | 81 | 0.016 |
| 147 | B*57 | 5 | 4 | 6 | 83 | 0.001 |
| 148 | A*74g | 4 | 5 | 8 | 58 | 0.035 |
| 148 | B*57 | 5 | 4 | 4 | 60 | 0.001 |
| 148 | B*5801 | 4 | 5 | 6 | 58 | 0.017 |
| 149 | B*1510 | 4 | 14 | 3 | 77 | 0.02 |
| 149 | B*4501 | 4 | 14 | 5 | 75 | 0.056 |
| 149 | B*57 | 6 | 12 | 5 | 75 | 0.004 |
| 149 | Cw*  030402 | 4 | 14 | 3 | 80 | 0.018 |
| 156 | A*0205 | 3 | 3 | 1 | 70 | 0.001 |
| 156 | B*5801 | 3 | 3 | 8 | 64 | 0.034 |
| 159 | A*2301 | 3 | 1 | 9 | 57 | 0.014 |
| 159 | B*4901 | 2 | 2 | 4 | 63 | 0.033 |
| 160 | A*0301 | 3 | 7 | 6 | 80 | 0.05 |
| 160 | Cw*02 | 5 | 5 | 17 | 72 | 0.041 |
| 169 | A*3001 | 7 | 25 | 4 | 63 | 0.035 |
| 169 | B*0702 | 7 | 25 | 1 | 66 | 0.001 |
| 169 | B*4201 | 20 | 12 | 4 | 63 | <0.0001 |
| 169 | B*47 | 2 | 30 | 0 | 67 | 0.02 |
| 169 | Cw*17 | 17 | 15 | 7 | 62 | <0.0001 |
|  |  |  |  |  |  |  |
